# FAN1 modifies Huntington’s disease progression by stabilizing the expanded *HTT* CAG repeat

**DOI:** 10.1093/hmg/ddy375

**Published:** 2018-10-24

**Authors:** Robert Goold, Michael Flower, Davina Hensman Moss, Chris Medway, Alison Wood-Kaczmar, Ralph Andre, Pamela Farshim, Gill P Bates, Peter Holmans, Lesley Jones, Sarah J Tabrizi

**Affiliations:** 1 UCL Huntington’s Disease Centre,Department of Neurodegenerative Disease, Queen Square Institute of Neurology, University College London,Queen Square, London WC1N 3BG, UK; 2 Medical Research Council Centre for Neuropsychiatric Genetics and Genomics, Cardiff University, CF24 4HQ, UK; 3 UK Dementia Research Institute, University College London, WC1N 3BG, UK

## Abstract

Huntington’s disease (HD) is an inherited neurodegenerative disease caused by an expanded CAG repeat in the huntingtin (*HTT*) gene. CAG repeat length explains around half of the variation in age at onset (AAO) but genetic variation elsewhere in the genome accounts for a significant proportion of the remainder. Genome-wide association studies have identified a bidirectional signal on chromosome 15, likely underlain by FANCD2- and FANCI-associated nuclease 1 (*FAN1*), a nuclease involved in DNA interstrand cross link repair. Here we show that increased *FAN1* expression is significantly associated with delayed AAO and slower progression of HD, suggesting *FAN1* is protective in the context of an expanded *HTT* CAG repeat. FAN1 overexpression in human cells reduces CAG repeat expansion in exogenously expressed mutant *HTT* exon 1, and in patient-derived stem cells and differentiated medium spiny neurons, FAN1 knockdown increases CAG repeat expansion. The stabilizing effects are FAN1 concentration and CAG repeat length-dependent. We show that FAN1 binds to the expanded *HTT* CAG repeat DNA and its nuclease activity is not required for protection against CAG repeat expansion. These data shed new mechanistic insights into how the genetic modifiers of HD act to alter disease progression and show that FAN1 affects somatic expansion of the CAG repeat through a nuclease-independent mechanism. This provides new avenues for therapeutic interventions in HD and potentially other triplet repeat disorders.

## Introduction

Huntington’s disease (HD) is a dominantly inherited neurodegenerative condition caused by expansion of a CAG trinucleotide repeat in the huntingtin (*HTT*) gene (1). The CAG region translates into an expanded polyglutamine stretch close to the N-terminus of the HTT protein which confers a toxic gain of function. Longer CAG repeats cause more severe disease, with earlier onset and faster progression (2). CAG repeats are unstable and prone to expansion during intergenerational germline transmission (gametic instability) and in somatic tissues as they age [somatic instability (SI)]. This is important because expansion is likely to result in an HTT protein with a longer and more toxic polyglutamine tract. Therefore, SI is likely to play an important role in HD pathogenesis, increasing with age in the tissues most affected by HD, particularly the striatum, and correlating with disease onset (3). Despite the correlation of *HTT* CAG repeat length with disease course, onset can still differ by several decades in patients with the same CAG repeat length (4,5). CAG repeat length accounts for ∼56% of variation in onset (4), but up to half of the remaining variability is heritable and therefore due to genetic differences elsewhere in the genome (6).

Recent genome-wide association studies (GWASs) have identified genetic variation that influences HD age at onset (AAO) at a chromosome 15 locus that includes the FANCD2 and FANCI associated nuclease 1 (*FAN1*) gene with at least two independent signals, one advancing and the other delaying onset (7). These data imply polymorphisms in *FAN1*, a DNA repair enzyme, could be driving the GWAS signals (7), reinforced by a pathway analysis that showed DNA repair genes were associated with disease onset. This suggests that FAN1 may be part of a DNA damage response (DDR) network that modulates HD pathogenesis (7). Recent evidence demonstrates *Fan1* protects against expansion of the CGG repeat tract in the *Fmr* gene in a mouse model of Fragile X (8). A similar stabilization of the *HTT* CAG repeat tract would reduce somatic expansion and could underlie the effect of FAN1 on HD course.

**Table 1:** FAN1 expression is associated with slowed HD progression and delayed AAO

ID	CHR	Pos (Mb)	TWAS.Z (GeM)	TWAS.P (GeM)	TWAS.Z (track +registry)	TWAS.P (track +registry)
OCA2	15	28.0	0.97	3.30E-01	1.19	2.34E-01
HERC2	15	28.4	0.85	3.95E-01	2.70	6.90E-03
HERC2P9	15	28.9	−1.21	2.28E-01	1.13	2.59E-01
NDNL2	15	29.6	0.07	9.41E-01	1.09	2.76E-01
FAN1	15	31.2	6.99	2.80E-12	−3.78	1.58E-04
CHRNA7	15	32.3	0.10	9.20E-01	−0.01	9.93E-01
ARHGAP11A	15	32.9	−1.49	1.35E-01	0.10	9.21E-01
GREM1	15	33.0	−0.74	4.62E-01	−0.25	8.01E-01
AVEN	15	34.2	−0.48	6.28E-01	0.17	8.67E-01
CHRM5	15	34.3	0.84	4.03E-01	−1.35	1.78E-01
SLC12A6	15	34.5	0.77	4.43E-01	1.57	1.16E-01
NOP10	15	34.6	−0.29	7.74E-01	−1.35	1.78E-01
GOLGA8A	15	34.7	1.66	9.75E-02	−0.56	5.75E-01
GOLGA8B	15	34.8	0.46	6.49E-01	0.64	5.24E-01
AQR	15	35.1	1.01	3.13E-01	−1.76	7.92E-02
ZNF770	15	35.3	0.93	3.54E-01	−0.26	7.92E-01

TWAS results from a 5 Mb either side of FAN1 using predictors derived from Common Mind Consortium prefrontal cortex expression data applied to summary statistics from the GeM study of AAO and the TRACK+REGISTRY study of progression. Positive Z-scores indicate that increased expression associates with later AAO (GeM) or faster progression. Genes with nominally significant TWAS association are highlighted in red.

Functional redundancy is common in the DDR, with components participating in multiple independent pathways (9,10). Interaction between mismatch repair (MMR) and interstrand cross link (ICL) DNA repair pathways has been reported (10), with FAN1 capable of compensating for loss of EXO1 MMR activity under some circumstances (11). Therefore, FAN1 and MMR components may modulate HD AAO through a shared mechanism. A stable physical interaction between FAN1 and MutLα components MLH1 and PMS2 further supports this hypothesis (12). FAN1 is a DNA endo/exonuclease involved in DNA repair that is highly expressed in the brain (12,13). It was originally identified as a component in the Fanconi anemia (FA) ICL repair pathway (12,13–16), though its loss does not cause FA but karyomegalic interstitial nephritis, a rare recessive kidney disease caused by loss of FAN1 activity (17–19). FAN1 also regulates genomic stability and the recovery of stalled replication forks independent of the FA pathway (20,21). These functions require FAN1 nuclease activity. FAN1 co-migrates in a complex with MLH1 and PMS2 that form MutLα, suggesting this complex plays an important but as yet unidentified role in FAN1 function. This is pertinent because evidence from mouse models suggests MMR components are required for SI (22,23), and pathway analysis highlights DNA MMR as a strong driver of HD pathogenesis (7). Two MMR components, *MSH3* and *MLH1*, were recently identified as modulators of HD progression and AAO, respectively (24,25). As both FAN1 and MMR regulate DNA repeat stability (8,26,27), interactions between these components suggest they may contribute to a common pathway.

In this study, we find that increased *FAN1* expression is significantly associated with delayed AAO and slower disease progression in HD patients. We show FAN1 expression profoundly suppresses CAG repeat expansion in the U20S cell line expressing mutant *HTT* exon 1, and knockdown (KD) of *FAN1* expression accelerates CAG repeat expansion in HD patient-derived induced pluripotent stem cells (iPSCs) and differentiated medium spiny neurons (MSNs). Further, we show this stabilization is FAN1 concentration-dependent and does not depend on its nuclease activity. Although FAN1 binds to CAG repeat DNA, we do not find it is targeted specifically to the expanded repeat sequences. We propose that FAN1 modulates HD pathogenesis and stabilizes the *HTT* CAG repeat region by acting in concert with other DDR proteins. Understanding the mechanism by which DNA repair components influence disease course may provide tractable therapeutic targets for HD.

## Results

### FAN1 has a protective role in HD

A transcriptome-wide association study (TWAS) was performed to identify genes with expression significantly associated with altered HD AAO and progression (Table 1). The method of Gusev *et al.* (28) was used to impute gene expression values from 452 dorsolateral prefrontal cortex samples from the Common Mind Consortium (29) into the GeM Consortium (GeM) GWAS of AAO (7) and the TrackHD and Registry (track + registry) GWAS of HD progression (24,30). The Z-score represents the standardized effect size, with positive values indicating that increased expression is associated with later onset (GeM) or faster progression (track + registry). Assuming a Bonferroni correction for the number of genes in the TWAS (5261 genes for the Common Mind Consortium (CMC) cortex) would give a significance criterion of *P* < 9.5 × 10^−6^. Thus, increased FAN1 expression is significantly associated with delayed AAO of HD. In addition, FAN1 trends toward significance in the track cohort indicating decreased FAN1 expression is associated with earlier AAO and faster progression. That is, FAN1 expression is protective in the context of an expanded *HTT* CAG repeat in both cohorts. No other nearby genes reach nominal significance in both cohorts. Thus, our TWAS data clearly indicate that *FAN1* expression has a role in modifying HD onset and progression.

**Figure 1:**
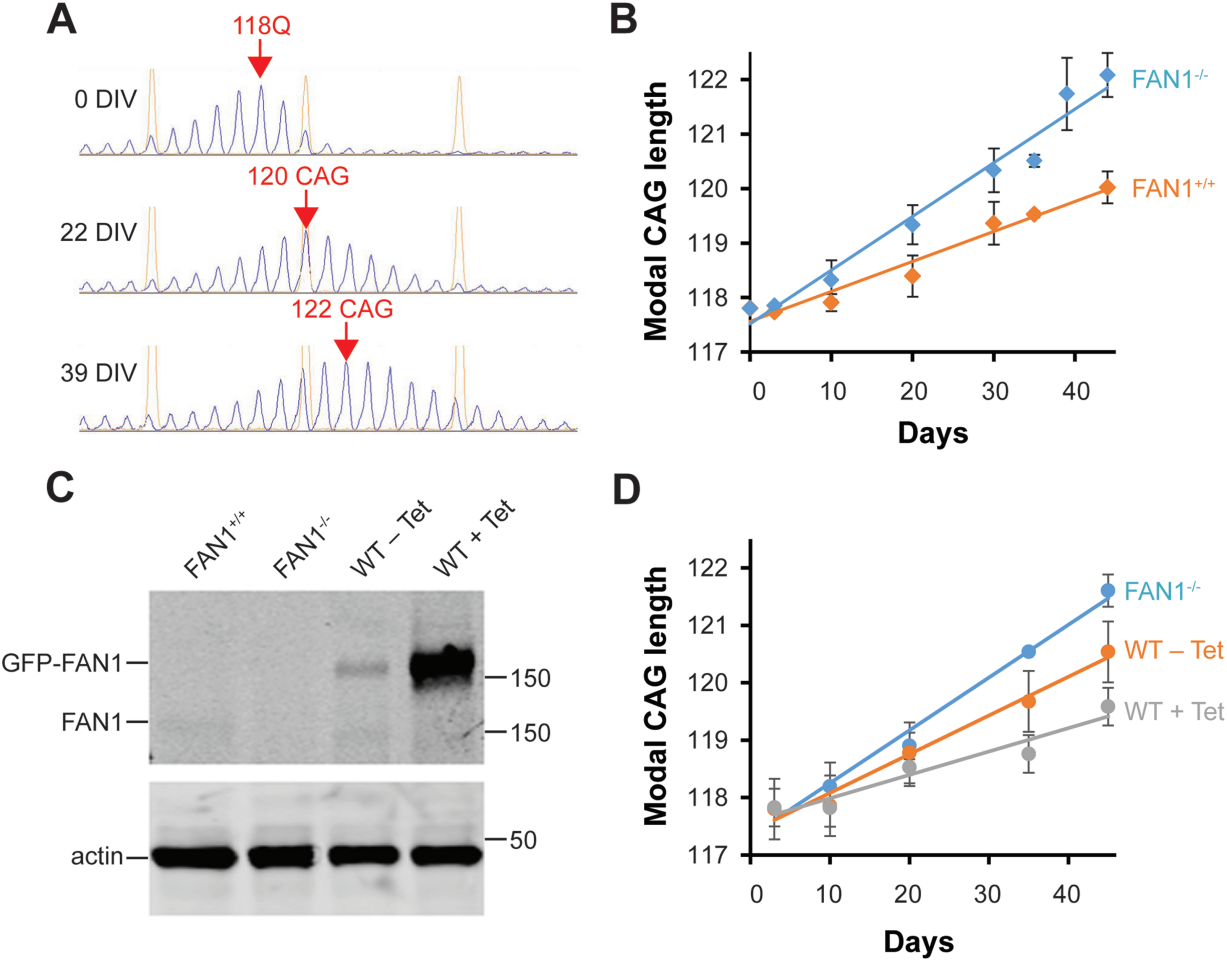
FAN1 expression stabilizes the *HTT* CAG repeat in culture. **(A)** Fragment analysis shows the change in modal CAG repeat size over time in U20S FAN1^−/−^ cells stably transduced with *HTT* exon 1 (118 CAG). Representative capillary electrophoresis traces are shown with modal CAG repeat length shown in red text**. (B)** Increase in modal CAG repeat size in FAN1^−/−^ and FAN1^+/+^ cells stably transduced with *HTT* exon 1 (118 CAG). Note that FAN1 expression (+/+) significantly slows CAG expansion rate (*P* = 0.000269, *n* = 6 for each condition). **(C)** Cell lysates were prepared for SDS-PAGE from U20S FAN1^+/+^, FAN1^−/−^ and cells reconstituted with FAN1^GFP-WT^, with or without induction by Tet (0.1 ug/ml). Immunoblots developed with FAN1 or actin antibodies are shown. In the absence of Tet, low levels of GFP–FAN1 are expressed, close to endogenous expression levels seen in FAN1^+/+^ cells. Tet induces high FAN1 expression. Note the reduced mobility of the GFP–FAN1 forms. **(D)** Modal CAG repeat size in FAN1^−/−^ or FAN1^GFP-WT^ cells (±Tet induction) stably transduced with *HTT* exon 1 (118 CAG). Data from three replicates for each FAN1^GFP-WT^ condition and six for FAN1^−/−^ are shown. Tet was included in three of the FAN1^−/−^ experiments shown in this panel but was found to make no significant difference to the CAG repeat expansion rate so the data were pooled. Note, expression of GFP–FAN1 (WT-Tet) significantly reduces CAG repeat expansion rate (*P* = 0.000257). Increasing GFP–FAN1 expression by Tet induction (WT + Tet) further slows CAG repeat expansion.

### FAN1 expression stabilizes the CAG repeat in U20S *HTT* exon 1 cells

To investigate how FAN1 expression might modify HD onset and progression, we introduced wild-type (WT) or variant *FAN1* isoforms in a tetracycline (Tet)-inducible green fluorescent protein (GFP) expression cassette into *FAN1* knockout U20S osteosarcoma cells (31) (FAN1^−/−^; Supplementary Material, Fig. S1). *HTT* exon 1 constructs with a range of CAG repeat lengths between 30 and 118 units were stably introduced into these cells by lentiviral transduction, allowing study of interactions between FAN1 and *HTT* CAG repeat DNA. A U20S line expressing endogenous FAN1 provides an additional control (FAN1^+/+^). GFP–FAN1 constructs were functional in ICL repair and bound known FAN1 interactors FANCD2, MLH1 and PMS2 (Supplementary Material, Fig. S1).

Firstly, we assessed CAG repeat size within the *HTT* exon 1 construct of our longest CAG repeat (118 CAG) over extended periods in culture using fragment analysis. This polymerase chain reaction (PCR)-based analysis allows accurate sizing of the CAG repeat. It typically results in a ‘hedgehog’ capillary electrophoresis trace with a series of peaks separated by one CAG unit in a roughly Gaussian distribution caused by natural variation within the sample and PCR stutter (Fig. 1A). The tallest peak represents the most common or modal value, usually taken as the CAG repeat size for a given sample. U20S FAN1^−/−^*HTT* exon 1 (118 CAG) showed a smooth, apparently linear increase in the modal CAG length (Fig 1B). An average increase in 1 CAG unit increase per 10.1 ± 0.8 days was observed. The expression of endogenous FAN1 significantly slowed repeat expansion (FAN1^+/+^: 1 CAG unit increase per 17.7 ± 1.4 days; *P* = 0.000269 compared to FAN1^−/−^ cells; Fig. 1B). Cells expressing GFP–FAN1 at near endogenous levels (Fig. 1C) were similarly stabilized (FAN1^WT^ − Tet: 1 CAG unit increase per 14.8 ± 1.1 days; *P* = 0.000257 compared to FAN1^−/−^ cells; Fig. 1D). Tet-induced overexpression of GFP–FAN1 (Fig. 1C) further decreased the CAG repeat expansion rate (FAN1^WT^ + Tet: 1 CAG unit increase per 21.3 ± 1.8 days; *P* = 0.0806 compared with FAN1^WT^ − Tet cells; Fig. 1D). These data show increasing FAN1 expression correlates with a slowed CAG expansion rate.

**Figure 2:**
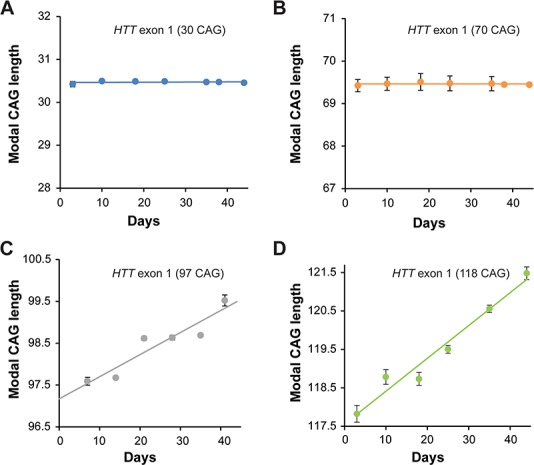
*HTT* CAG repeat stability in culture is length dependent. (**A–D)** Modal CAG repeat size in FAN1^−/−^ cells stably transduced with the indicated *HTT* exon 1 constructs. Repeats with 30 CAG and 70 CAG are stable over the culture period of 40 days. The 118 CAG repeats are unstable as expected. The 97 CAG repeats expand but at a significantly slower rate than the longer 118 CAG repeat (*P* = 1.38 × 10^−5^). Data from three replicates per condition are shown.

To study the CAG length dependency of the repeat sequence, a series of *HTT* exon 1 constructs containing 30, 70 or 97 CAGs was introduced into FAN1^−/−^ cells. The 30 CAG repeat, which is a non-pathogenic length, and 70 CAG repeat tracts were stable in culture over 8 weeks (Fig. 2). This was consistent with observations of long-term culture of an iPSC line derived from an HD patient with 73 CAG (Supplementary Material, Fig. S2A). U20S FAN1^−/−^*HTT* exon 1 (97 CAG) cells showed repeat expansion, although the average rate of increase was slower than that observed in the 118 CAG line (1 CAG unit increase per 18.3 ± 2.2 days; *P* = 1.38 × 10^−5^ compared to 118 CAG; Fig. 2). These data suggest that CAG repeat expansion in cultured cells is detectable over this short period if long CAG repeat lengths are used and also demonstrate that the rate of CAG expansion is length-dependent.

### FAN1 nuclease inactivation and the p.R507H polymorphism do not affect *HTT* CAG repeat stability in U20S cells

**Figure 3:**
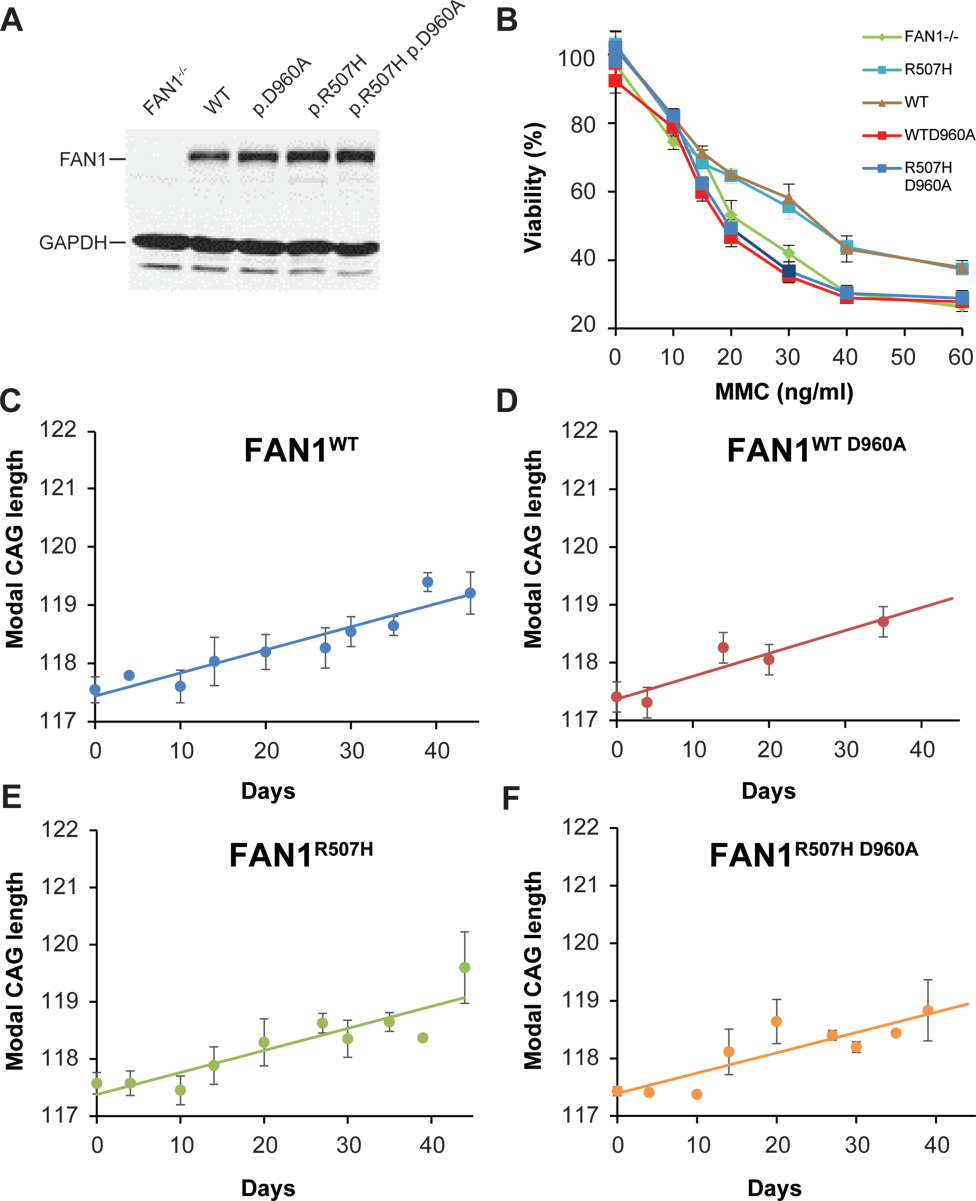
FAN1 nuclease activity and p.R507H polymorphism do not influence *HTT* CAG repeat stability. **(A)** Immunoblot showing FAN1 levels in U20S cells reconstituted with catalytically active or nuclease-dead WT or p.R507H variant FAN1 forms. The FAN1^−/−^ line is shown for comparison. Note that the p.R507H variant and p.R507H D960A forms are expressed at a higher level**. (B)** U20S cell lines were treated with MMC at various concentrations for 16 h. The cells were washed into fresh media and viability was assessed after 10 days in culture. FAN1^−/−^ and nuclease-dead mutant lines are equally vulnerable to MMC toxicity. Catalytically active FAN1 forms infer protection. Data from three replicates per condition are shown. **(C–F)** Modal CAG repeat size in FAN1^−/−^ cells reconstituted with FAN1 isoforms, as indicated, and stably transduced with *HTT* exon 1 (118 CAG); linear trend lines have been fitted to the data. CAG expansion rate did not differ between WT, p.R507H, p.D960A (nuclease-dead) or the combined p.R507H + p.D960A cell lines. Data from three (p.R507H forms) or four (WT forms) replicates per condition are shown.

To investigate which aspects of FAN1 function regulate CAG repeat stability in the U20S cells, FAN1 variants were introduced into the WT GFP–FAN1 construct by site-directed mutagenesis; the p.D960A mutation inactivates the nuclease domain (14,15), and the p.R507H variant within the DNA-binding domain was the most significant coding small nuclear polymorphism in *FAN1* from the GeM HD GWAS (7). Western blot analysis confirmed protein expression in FAN1^−/−^ cells reconstituted with these FAN1 forms but showed that FAN1 levels varied. Quantification of immunoblots showed that the p.R507H variants were expressed at 1.2–1.5 times the levels of the WT protein (Fig. 3A). U20S cells expressing either WT or p.R507H *FAN1* were fully functional in ICL repair (Fig. 3 and Supplementary Material, Fig. S1). Consistent with this, lymphoblastoid (LB) cells derived from patients with WT or p.R507H FAN1 also showed equivalent ICL repair activity (Supplementary Material, Fig. S1). As expected, FAN1^−/−^ and the nuclease-inactivated p.D960A mutation, either on a WT or p.R507H FAN1 background, increased mitomycin C (MMC) sensitivity, indicative of compromised ICL repair function (Fig. 3B).

These cells were transduced with the 118 CAG *HTT* exon 1 construct, and the stability of the trinucleotide repeat was assessed (Fig. 3C–F). There was no significant difference in CAG expansion rate in cells expressing the WT or nuclease-dead D960A mutant (*P* = 0.331 FAN1^WT^ compared with FAN1^WT D960A^). CAG repeat expansion was significantly slower in both lines than FAN1^−/−^ cells transduced in parallel (FAN1^WT^ and FAN1^WT D960A^*P* = 1.96 × 10^−8^). Similarly, expression of the D960A mutation on a p.R507H background did not affect expansion rate relative to the catalytically active p.R507H isoform (*P* = 0.882, FAN1^p.R507H^ compared with FAN1^p.R507H D960A^). These *FAN1* forms also slowed CAG expansion relative to FAN1^−/−^ cells (FAN1^p.R507H^*P* = 2.08 × 10^−11^ and FAN1^p.R507H D960A^*P* = 1.58 × 10^−9^). There was a trend toward slower CAG repeat expansion in cells expressing the p.R507H *FAN1* isoform (FAN1^p.R507H^*P* = 0.0692 relative to FAN1^WT^). These results suggest that the nuclease domain is not required for FAN1 to stabilize the *HTT* CAG repeat and that the p.R507H variant does not significantly increase CAG repeat expansion rate.

### FAN1 expression regulates the stability of the endogenous CAG repeat in *HTT*

To assess the stability of the CAG repeat in the endogenous *HTT* gene, we used iPSCs derived from an HD patient with 109 CAG repeats (32). At baseline, the CAG repeat was measured at 121 units, indicating expansion from the original length, and during characterization of the cells over 58 passages the repeat expanded to 131 units. Expansion in long-term culture fits to an exponential model (r2 = 0.99, *P* = 7.65 × 10^−26^) (Supplementary Material, Fig. S2B). Current cells have been in culture for 300 days, have a CAG length of 147 repeats, expand at a rate of 1 CAG repeat every 12.4 days and would be predicted to reach 200 repeats in 19 months.

**Figure 4:**
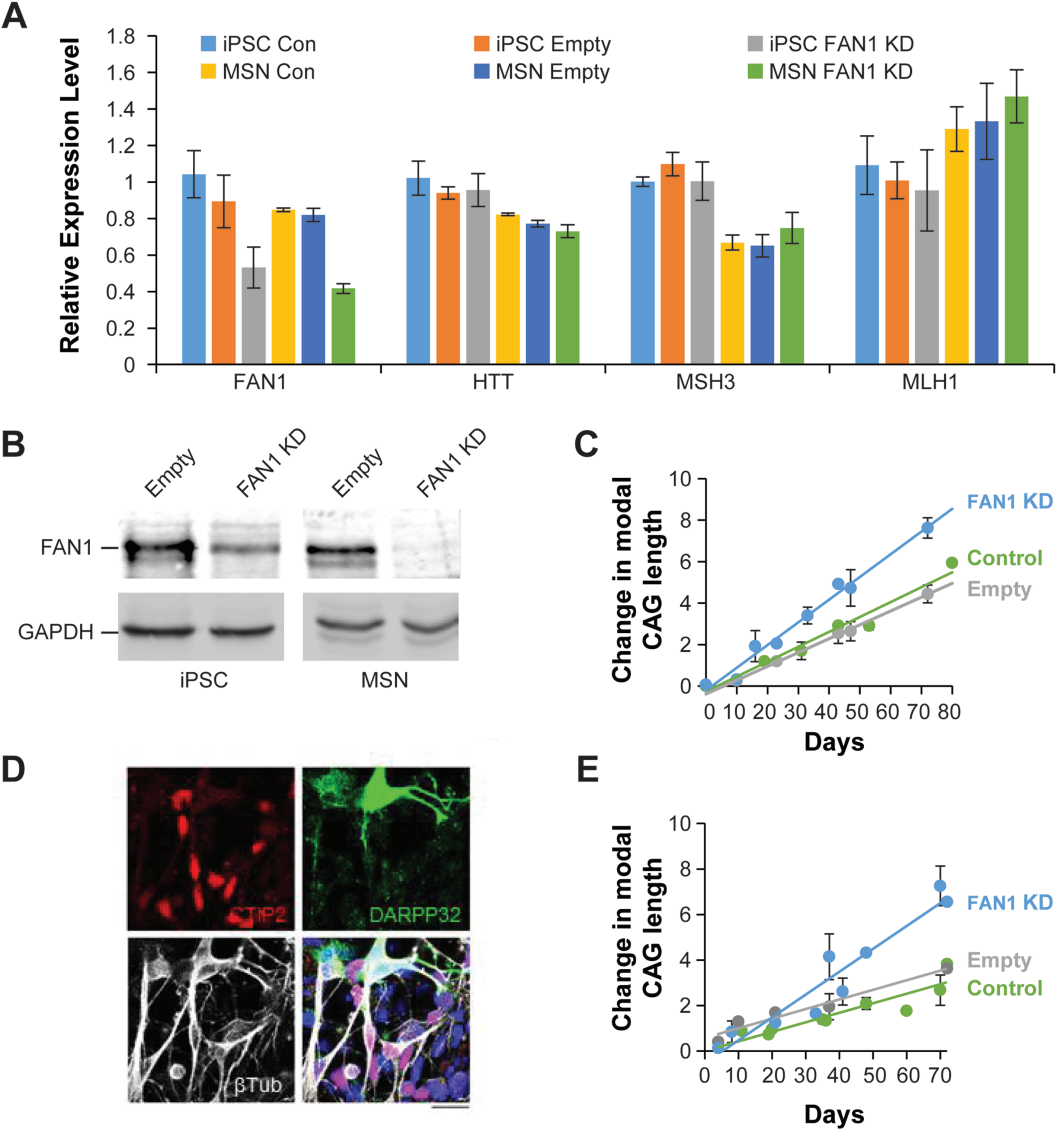
FAN1 expression regulates the stability of the endogenous CAG repeat in *HTT* gene. **(A)** qPCR showing gene expression in the 109 CAG iPSC line and differentiated MSNs derived from this line following stable introduction of an shRNA targeting *FAN1.* Control cells and empty vector-treated cells are also shown. Expression of *FAN1* was significantly reduced by the introduction of shRNA (53.1% KD in iPSCs, *P* = 0.017), whereas transcript levels of *HTT, MSH3* and *MLH1* were unchanged. FAN1 KD was maintained throughout differentiation. MSN cells were harvested at day 36 on the scale shown in panel (E) for this analysis. Data from three independently transduced or mock-treated (control) lines are shown. **(B)** Western blot shows FAN1 KD in the 109 CAG iPSC line following stable introduction of an shRNA targeting *FAN1.***(C)** Change in modal CAG repeat size over time is shown for control 109 CAG iPSC cells (green) or cells treated with shRNA targeting *FAN1* (blue) or control empty vector (gray). Note the increased rate of expansion in the cells lacking FAN1. Data points represent mean ± SEM from three iPSC lines transduced independently for each treatment group. Linear trend lines have been fitted to the data. Statistical analysis indicated significant difference in expansion rate between FAN1 KD and control (*P* = 1.579 × 10^−8^). **(D and E)** SI of the CAG repeat region from the iPSC lines with 109 CAG continues in post-mitotic neurons. (D) Confocal image of 109 CAG cell line following MSN differentiation. The images were taken at day 36 on the scale shown in panel (E). Note the expression of DARPP32 (green), MSN marker CTIP2 (red) and neuronal marker βIII tubulin. DAPI (blue) is included in the merged image (scale bar 20 μm). (E) Change in CAG repeat length over time. FAN1 KD accelerates CAG expansion in differentiating cells. Data points represent mean ± SEM from three iPSC lines transduced independently for each treatment group. Linear trend lines have been fitted to the data. Statistical analysis indicated significant difference in expansion rate between FAN1 KD and control (*P* = 6.15 × 10^−7^).

To verify the effects of FAN1 on the CAG repeat region in the endogenous *HTT* gene, we introduced shRNA targeting *FAN1* into this cell line using retroviral transduction. *FAN1* expression was reduced at both mRNA and protein levels (Fig. 4A and B). FAN1 KD was maintained over the period of the experiment (Fig. 4A and B). Expression of *HTT* and MMR components *MLH1* and *MSH3* were not affected (Fig. 4A).

The CAG repeat increased in size in the control and empty vector-treated cells at a similar rate (1 CAG unit every 17.7 ± 1.1 and 16.1 ± 1.2 days; *P* = 0.573). However, *FAN1* KD significantly increased expansion rate to 1 repeat every 9.1 ± 0.5 days (Fig. 4C; *P* = 7.81 × 10^−7^ as compared to untreated and empty vector-treated cells). These results support data from U20S cells, suggesting that FAN1 protects against expansion of the endogenous *HTT* CAG repeat, at least in mitotic cells.

To assess if this mechanism also operates in non-dividing cells, we used an established differentiation protocol to generate MSNs from control and retrovirally transduced iPSC lines (33). Significantly increased expression of a panel of MSN markers, including BCL11B (CTIP2), DRD1, DRD2, GAD2, TAC1, OPRM1, PENK and CALB1, as well as the neuronal markers DARPP32 and βIII relative to iPSCs, demonstrates that a high proportion of mature striatal neurons were generated by day 36 of this protocol (Fig. 4D and Supplementary Material, Fig. S3). Serial cultures were grown and harvested during differentiation and as the MSNs age up to day 70. Western blot and quantitative PCR (qPCR) showed that *FAN1* KD was maintained throughout differentiation (Fig. 4A and B). Fragment analysis showed that CAG expansion rate was equivalent in the untreated differentiated MSNs and untreated iPSCs (1 repeat every 17.7 ± 1.1 and 16.7 ± 2.3 days). CAG expansion rate was not significantly different in untreated and empty vector-treated MSNs (1 repeat every 23.7 ± 2.8 days for the latter; *P* = 0.661). Critically, *FAN1* KD significantly increased CAG expansion rate to 1 CAG repeat every 9.1 ± 0.6 days (*P* = 0.00167 compared to control and empty vector-treated cells).These data suggest that FAN1 restrains CAG repeat expansion in cultures that contain a high proportion of differentiated post-mitotic striatal neurons (Fig. 4).

### FAN1 associates with the *HTT* CAG repeat

FAN1 interactions with CAG repeat DNA in *HTT* exon 1 were assessed using chromatin immunoprecipitation (ChIP) assays. Initially FAN1^−/−^, GFP–WT- or GFP–p.R507H-expressing U20S cells were transiently transfected with the *HTT* exon 1 fragment containing 118 CAG repeats to introduce high copy numbers of the plasmid DNA. FAN1 expression was maximized by Tet induction, optimizing conditions for detecting an interaction with the *HTT* exon 1 DNA. This system permits pull down of GFP–FAN1 forms and associated DNA using highly specific and efficient GFP-Trap beads (ChromoTech, Germany). ChIP fractions were probed by PCR with primers spanning the CAG repeat. Low but reproducible levels of CAG repeat DNA were detected in the ChIP fractions from cells expressing both GFP–FAN1 WT and p.R507H variant forms (Supplementary Material, Fig. S4A). ChIP fractions from the control FAN1^−/−^ cells did not contain detectable DNA, demonstrating the specificity of the ChIP pull down. These results suggest a novel interaction between FAN1 and CAG repeat DNA.

**Figure 5:**
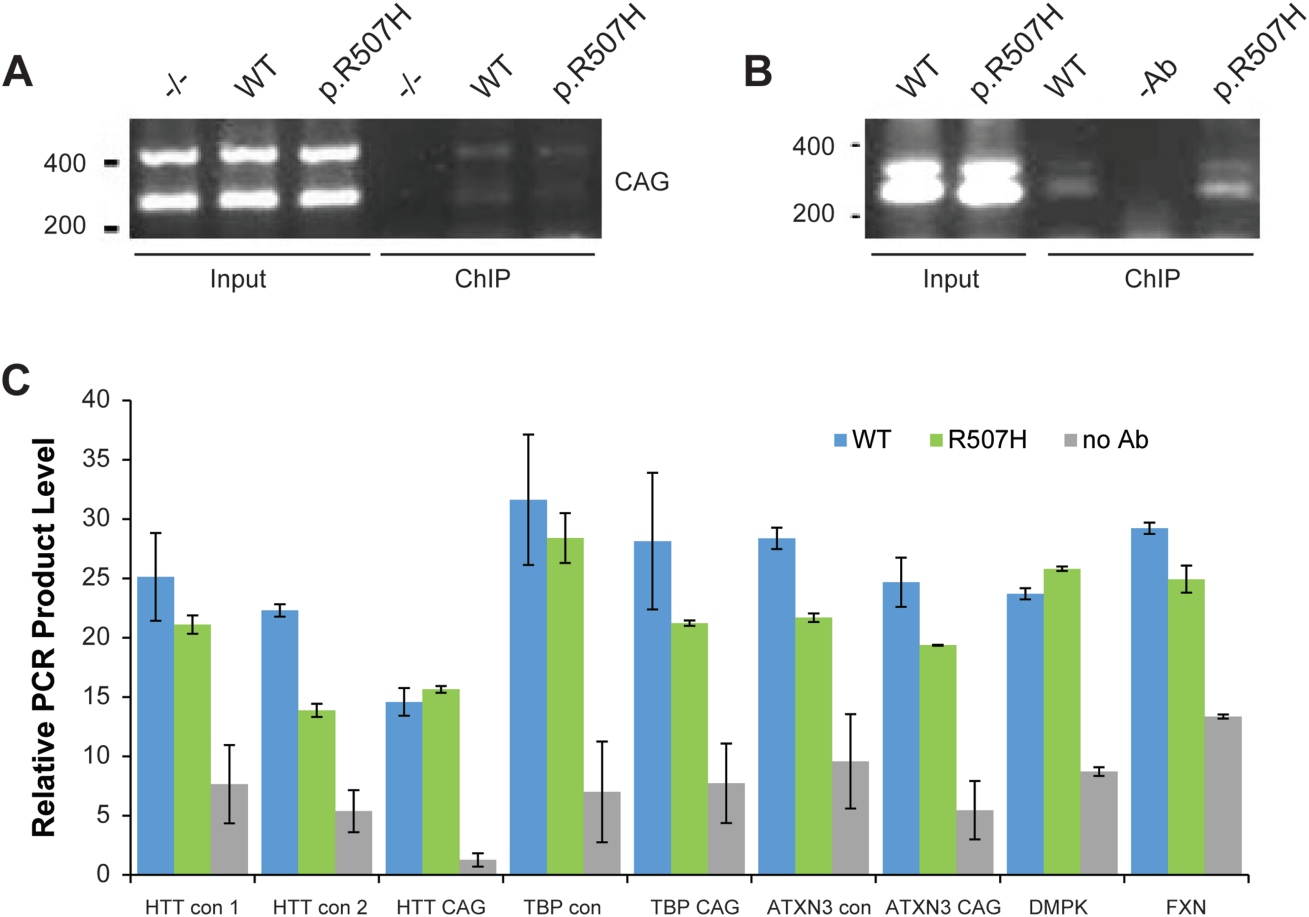
FAN1 associates with the *HTT* CAG repeat. **(A)** U20S *HTT* exon 1 70 CAG FAN1 knockout (−/−) cells or cells reconstituted with WT or p.R507H proteins were prepared for ChIP. GFP-Trap beads were used to isolate FAN1 forms and associated DNA. Agarose gel showing input (5%) and ChIP fractions amplified with primers spanning the *HTT* CAG repeat. The two bands visualized using CAG primers in the input and ChIP fractions are DNA amplified from the introduced *HTT* exon 1 plasmid (70 CAG units) and DNA from the endogenous *HTT* gene (20 CAG units). ChIP fractions isolated FAN1^−/−^ cells did not contain detectable amounts of DNA, indicating the specificity of the ChIP procedure**. (B)** Extracts from HD LB cells homozygous for WT FAN1 or heterozygous for the p.R507H variant were prepared for ChIP analysis, and a FAN1 antibody was used to isolate FAN1 and associated DNA. Mock pull downs with no antibody were used as controls. Primers spanning the CAG repeat of the *HTT* gene were used to amplify isolated DNA. PCR products were visualized on an agarose gel. Input (5%) and ChIP fractions are shown. The two bands seen in the input and ChIP fractions are amplified DNA from normal and mutant expanded *HTT* alleles. Controls without antibody contained little DNA showing the specificity of the ChIP experiment. **(C)** ChIP fractions shown in (B) were amplified with primer pairs targeting different regions of *HTT* (CAG repeat and non-CAG regions—HTT con 1 proximal to the repeat and 2 targeting the 5′ end of *HTT*) and other CAG repeat-containing genes regions. The data indicate that FAN1 is not specifically targeting the *HTT* CAG repeat region. The mean from three independent experiments is shown (error bars = standard deviation).

We then made use of our panel of U20S cells expressing GFP-WT or GFP–p.R507H FAN1 and stably transduced with *HTT* exon 1 of 70, 97 and 118 CAG repeat lengths. The *HTT* CAG fragments are integrated and transcriptionally active (Supplementary Material, Fig. S4B and C). Again GFP-Trap beads were used to pull down GFP–FAN1 forms, with corresponding FAN1^−/−^ cells acting as controls. Primers spanning the *HTT* CAG repeat amplified low levels of DNA from ChIP fractions isolated from U20S *HTT* exon 1 cells with 70 CAG repeats. In addition, these ChIP fractions contained amplified DNA derived from the endogenous, non-pathogenic *HTT* gene (20 CAG repeat units), as visualized as a doublet on an agarose gel (Fig. 5A). As expected, ChIP fractions from control FAN1^−/−^ cells did not contain detectable levels of DNA. Similar levels of PCR product were generated from ChIP fractions derived from WT and p.R507H variant FAN1 forms. No clear differences in the levels of the shorter (WT allele) or longer (introduced exon 1 DNA) CAG repeat DNA could be discerned.

To demonstrate that FAN1 interacts with expanded CAG repeat DNA of the endogenous *HTT* gene we utilized LB cells from two HD patients with similar FAN1 expression levels, one homozygous for WT *FAN1* and the other heterozygous for the p.R507H variant (Supplementary Material, Fig. S1). To isolate ChIP fractions, a highly specific FAN1 antibody was used (Supplementary Material, Fig. S1). The mutant *HTT* allele in these cells contains 42 and 43 CAG repeats. Input and ChIP fractions were isolated and probed with the primers spanning the CAG repeat. PCR products are visualized as a doublet on the agarose gel, most clearly seen in the input fractions (Fig. 5B). ChIP fractions from both WT and p.R507H *FAN1* cell lines contained low but reproducible levels of PCR product derived from both long and short *HTT* alleles, indicating that FAN1 interacts with both the normal and mutant *HTT* CAG repeats. Both contained similar levels of PCR product (Fig. 5B). Control ChIP reactions in which the FAN1 antibody was omitted contained low background levels of CAG repeat DNA.

To determine the specificity of FAN1’s interaction with *HTT* CAG repeat DNA, we quantified the DNA fragments isolated in the FAN1 ChIP fractions by qPCR. Together with the primers spanning the CAG repeat, pairs targeting two regions of *HTT*, one proximal to the CAG repeat region and one distal at the 3′ end of the gene, along with others targeting different genes with CAG repeat regions and other control genes, were used to probe the ChiP DNA (Fig. 5C). We found no significant enrichment for the *HTT* CAG repeat or indeed any of the CAG repeat DNA from other genes (Fig. 5C). Thus, FAN1 is not detectably targeted to the CAG repeat region of *HTT*.

We performed similar experiments using the 109 CAG iPSCs, which are homozygous for WT *FAN1* and containing an unstable *HTT* CAG repeat (Supplementary Material, Fig. S4). ChIP fractions isolated using the FAN1 antibody contained DNA amplified from both long and short *HTT* alleles with no obvious differences in amount detectable. qPCR showed the ChIP fractions contained similar levels of DNA amplified with primers spanning the CAG repeat or control regions of *HTT* (Supplementary Material, Fig. S4).

## Discussion/Conclusion

Recent GWAS have identified a locus on chromosome 15, likely underlain by *FAN1*, as a modifier of HD onset (7). Through a TWAS, we demonstrate that increased *FAN1* expression is significantly associated with delayed HD onset and slower disease progression. This suggests that FAN1 is protective in the context of an expanded *HTT* CAG repeat. We used a series of cell models to show that WT FAN1 restrains CAG repeat expansion in both dividing and post-mitotic HD MSNs. Expanded *HTT* CAG repeats are somatically unstably *in vivo*, and CAG repeat expansion correlates with tissue-specific neuropathology and disease severity (3). Our data suggest that increased FAN1 expression delays HD onset and slows progression by restricting CAG somatic expansion.

*FAN1* is the only gene significant in GeM TWAS and trends toward significance in the TRACK cohort (Table 1), suggesting its expression level in cortex influences HD phenotype. While it is possible that other genes at the chromosome 15 locus, such as *MTMR10*, are driving the GWAS signal, the data presented here strongly support FAN1 as the HD modifier.

Recent evidence that FAN1 restricts GCC trinucleotide repeat DNA expansion in a mouse model of Fragile X is consistent with our data (8). This supports our hypothesis that FAN1 modulates HD course by stabilizing the *HTT* CAG repeat. FAN1 is likely to play a key role in a network of DDR proteins, as suggested by GWAS pathway analyses that find significant association of DNA repair gene sets independent of FAN1 (7,24,25). These pathways include MMR components MSH3 and MLH1; inactivation of which in HD mouse models abrogates somatic expansion and ameliorates the HD phenotype (22,23). Our *in vitro* studies suggest that FAN1 also contributes to this mechanism, protectively inhibiting CAG expansion.

Repeat instability likely involves the formation of unusual DNA secondary structures such as slipped strands, hairpin loops, G-quadruplexes and R-loops during DNA transcription, replication and repair (34). The high complementarity of the repeating sequence can lead to slippage mutation or mishybridization (35). These features are recognized by DNA repair proteins, notably members of the MMR pathway and its co-factors, which may trigger attempts at repair that lead to the incorporation of additional CAG units. Longer CAG repeats are more unstable and prone to increased rates of expansion (35), a feature recapitulated in our U20S cells expressing *HTT* exon 1 with a series of CAG repeat lengths (Fig. 2). Over the short 40 day time course of our U20S assay, we observed a steadily increasing CAG repeat with a linear regression fitting our data closely. However, extended culture of 109 CAG iPSCs show CAG repeat expansion fits best to an exponential model, as would be expected (Supplementary Material, Fig. S2B). Patient cells with CAG repeat lengths typical of HD remain stable in culture. Even iPSCs and MSNs derived from a patient with a repeat of 73 CAG, a mutation causing juvenile onset HD, remained stable in culture for over 150 days (Supplementary Material, Fig. S2A). This may reflect the rarity of expansion events, particularly in shorter repeats, and it may be that instability can only be detected in these lines following significantly extended culture. In support of this, SI has been observed in mouse lines with similar CAG repeat sizes over a 9–24 month time frame (36,37). HD typically manifests in midlife and it has been suggested that this reflects slow somatic expansion of the CAG repeat over decades until a toxic threshold is reached, after which expression of a highly toxic polyglutamine tract, particularly in vulnerable tissues, causes disease (3,37,38).

**Figure 6:**
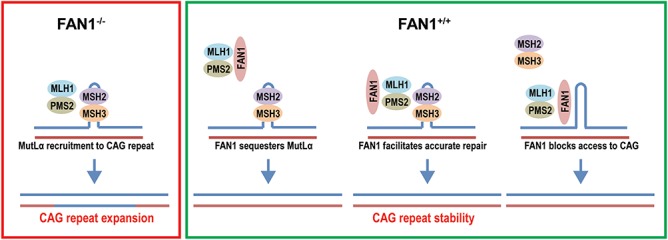
Mechanism of CAG repeat stabilization schematic showing a possible mechanism of FAN1 action. MutSβ (MSH2/MSH3) binds to abnormal structures formed by CAG repeat DNA, represented by a hairpin loop in the top strand. In the absence of FAN1 (purple box, FAN1^−/−^), MutLα (MLH1/PMS2) is recruited to the lesion by MutSβ. This leads to MMR activation, error-prone repair and CAG expansion. FAN1 expression (green box) may stability the CAG repeat by sequestering MutLα. This prevents its interaction with MutSβ thereby stopping MMR activation and CAG expansion. Alternatively, the association of FAN1 with the CAG repeat facilitates accurate repair, possibly by recruiting DDR components or FAN1 blocks access to the CAG repeat, preventing access to MMR components and CAG expansion.

The mechanism by which FAN1 exerts its protective effect is unknown but different models can be postulated (Fig. 6). FAN1, which is structure rather than sequence-specific (39), may bind abnormal DNA structures formed by the CAG repeat, physically blocking access of other DNA repair proteins and preventing error-prone repair. Alternatively, FAN1 may promote accurate repair of the CAG repeat, either directly or by acting as a scaffold for assembly of a protein complex. Our ChIP results suggest FAN1 binds at or very near the CAG repeat, possibly recognizing unusual conformations formed by this type of sequence (40). However, we were unable to demonstrate any enrichment for mutant CAG repeat DNA in the ChIP fraction, indicating that FAN1 does not preferentially interact with the expanded CAG sequence. In addition, the p.D960A mutation which inactivates FAN1’s nuclease activity did not impair its ability to stabilize the CAG repeat. This is known to be a null mutation, so even the high expression levels used in our U20S cell model will not generate any nuclease activity (as demonstrated in ICL repair assays; Fig. 3). This observation is unusual in FAN1 biology, with all functions identified to date depending on its nuclease activity (12,18–21,41). Our data suggest a novel FAN1 activity is regulating CAG stability.

The FAN1 interactome includes several proteins already known to affect CAG repeat stability, including MutL components (MLH1, MLH3 and PMS2) and proliferating cell nuclear antigen (PCNA) (12,41). FAN1 is recruited to stalled replication forks by ubiquitinated PCNA (41), but no function has yet been described for the interaction with MLH1, despite evidence indicating this complex is stable, and comprises a substantial proportion of the cellular FAN1 and MLH1 under steady-state conditions (12). MutLα components MLH1 and PMS2 are readily detectable in GFP-Trap pull down fractions from U20S cells expressing GFP–FAN1 (Supplementary Material, Fig. S1), whereas MutSβ does not bind to FAN1 (12). Therefore, FAN1 may sequester MLH1, preventing its interaction with MSH3 and the assembly of the MMR complex that promotes *HTT* CAG repeat expansion (Fig. 6).

The GeM HD GWAS suggest that the p.R507H *FAN1* variant is associated with earlier onset (7), so one would anticipate accelerated expansion in cells expressing it. U20S *HTT* exon 1 (118 CAG) cells expressing p.R507H showed reduced CAG repeat expansion rates compared to those expressing WT forms which trended toward significance. However, given the large effect size of FAN1 protein level (Figs 1 and 4), these changes are likely due to differences in *FAN1* expression rather than alteration of intrinsic protein function. It may be that our assay systems are not sensitive enough to detect small changes in activity that the p.R507H variant may engender. This is relevant because GWAS variants are often hypomorphic and the relatively high FAN1 levels expressed in the U20S cells could obscure subtle differences in activity between WT and p.R507H variant FAN1. This contrasts with the p.D960A mutation, which is known to have profound effects on FAN1 nuclease activity, compromising ICL repair activity in our U20S cell system to the level seen in FAN1^−/−^ cells (Fig. 3).

We have shown that the DNA repair protein FAN1 stabilizes the expanded *HTT* CAG repeat in cell models and patient-derived cells. Critically, this includes post-mitotic striatal MSNs generated from human HD iPSCs. These neurons model the region most vulnerable early in HD pathogenesis and that show the highest levels of SI (3,36,37). Therefore, they represent the most physiologically relevant *in vitro* model available to study SI. In the context of HD, FAN1 activity is predicted to reduce somatic expansion and thereby delay onset and slow disease progression. Our TWAS data are consistent with this hypothesis, suggesting that increased FAN1 expression delays onset and slows progression. DNA repair variants that modify disease course in HD also influence onset in other polyglutamine diseases (42), so modulation of FAN1 may have therapeutic potential in a range of neurodegenerative diseases caused by repeat expansion.

## Materials and Methods

### Transcriptome-wide association study

The method of Gusev *et al.* (28) was used to test for association between phenotype data from the GWAS summary statistics from the HD AAO in the GeM cohort (*n* = 4082) and HD progression in the combined track + registry cohort (*n* = 2078) and gene expression in control dorsolateral prefrontal cortex from the Common Mind Consortium (*n* = 452).

### Cell culture and manipulation

U20S SEC-C, HEK293T and Phoenix Ampho cells were grown in Dulbecco's minimal essential medium (DMEM) with GlutaMAX supplemented with fetal bovine serum (FBS) (10%) and pen/strep. FAN1 knockout and reconstitution were carried out as described previously (31). FAN1 mutants were generated by site-directed mutagenesis using the QuickChange XL kit according to the manufacturer’s instructions (Agilent, CA, USA). The presence of the DNA base changes was confirmed by sequencing of the genomic DNA isolated from reconstituted cells. The *HTT* exon 1 series with increasing CAG length was encoded in p’HRsincpptUCOE+htt IRES eGFP. Human *HTT* exon 1 lentiviral plasmids and related control vectors were previously described (43). This was transfected directly using GeneJuice for transient experiments or packaged in lentiviral particles for stable integration. HEK293T cells were transfected with packaging vectors and p’HRsincpptUCOE+htt IRES eGFP using Lipofectamine LTX. After 16 h, 8 ml fresh media was added. Cell media containing mature lentivirus was harvested 48 h post-transfection. This was filtered and frozen at −80°C or used directly.

For CAG expansion assays HEK293T-conditioned media containing p′HRsincpptUCOE+htt lentivirus was mixed one to one with fresh media supplemented with polybrene (8 μg/ml) and added to U20S cells for 24 h prior to a complete media change. At this point, Tet was added if appropriate and this was considered to be the start of each time course. Cells were grown to 80–90% confluence and sub-cultured 1:20 using (TrypZean, Sigma Aldrich, UK) to lift the cells. Excess cells were washed in phosphate buffered saline (PBS) and then frozen as a semi-dry pellet prior for genomic DNA extraction. The data shown are from three to six independent time course experiments. LB cells were grown in Roswell Park Memorial Institute (RPMI) supplemented with 15% FBS (non-heat inactivated), glutamine and pen/strep. For ICL repair assays, MMC was added to cells at increasing concentrations for 16 h. Cells were washed into fresh media and plated at 200 cells per well in a 96 well plate. Cells were cultured for 10–14 days and then the proportion of live cells was assayed. Cell survival was expressed as a percentage of control untreated cells. For acute MMC (200 ng/ml) or cisplatin (1 μg/ml) treatment drug was added for 2 h and then the cells were cultured in fresh media for 24–72 h prior to use. H2Ax foci clearance was assayed as described previously (12).

The shRNA hairpin targeting FAN1 (target sequence: GTAAGGCTCTTTCAACGTA) was subcloned into pSUPER.retro.Puro and transfected into Phoenix Ampho packaging cells using Lipofectamine LTX. After 16 h, 8 ml fresh media was added. Cell media containing mature retrovirus was harvested 48 h post-transfection. This was filtered and frozen at −80°C or used directly.

### iPSC culture and manipulation

Stem cells were maintained in Essential E8 medium (ThermoFisher) on Thermo-Nunc plasticware coated with Geltrex (Gibco) diluted 1:50 in DMEM/F12 without glutamine. They were passaged by manual dissociation using 0.02% EDTA (Gibco). MSN differentiation was carried out as described (32) using Activin A to direct ganglionic/striatal fate. Media containing retrovirus encoding shRNA hairpins targeting FAN1 or empty vector was mixed one to one with normal iPSC media and supplemented with polybrene (8 μg/ml). This media was added to iPSC at ∼70% confluence and the cells were incubated for 16 h. Fresh media was added to the cells for a further 48 h prior to selection. For this, the media was supplemented with puromycin (1 μg/ml) and the cells were monitored ensuring regular media changes to minimize the number of dead cells in the culture. Colonies of transduced cells were detected after 2–3 weeks. Untreated cells were cultured alongside the selected cells and used as controls in subsequent experiments.

### Fragment analysis ChIP and real-time qPCR

Fragment analysis was performed using standard methods with primer sequences detailed below. Samples were run on an ABI 3730 Genetic Analyser and analyzed using GeneMapper software (Thermo). For ChIP analysis cells were collected, washed in PBS and then cross linked with formaldehyde (1% final concentration) for 10 min at a room temperature. Excess formaldehyde was quenched by adding glycine to 125 mm and incubating for 5 min. Cross linked cells were pelleted, washed in PBS and frozen at −80°C or used directly. Cells were lysed in lysis buffer [15 mm Tris–HCL, pH 7.5, 0.3 m Sucrose, 60 mm KCl, 15 mm NaCl, 5 mm MgCl_2_, 0.1 mm EGTA, 0.5 mm DDT and 0.2% IGEPAL-CA supplemented with protease inhibitors] and nuclei were pelleted at 20 000 *g* for 20 min. Supernatants were aspirated and nuclei were resuspended in sonication buffer (50 mm Tris, pH 8.0, 10 mm EDTA and 1% sodium dodecyl sulphate (SDS) supplemented with protease inhibitors). Chromatin was fragmented by 10 cycles of 30 s sonication in a Bioruptor apparatus. Ice water was added to the sonication bath to ensure that temperature was regulated during disruption. Insoluble material was cleared by centrifugation at 20 000 *g* for 10 min. This sonicated fraction was used diluted 10-fold with dilution buffer (16.7 mm Tris pH 8.0, 1.2 mm EDTA, 167 mm NaCl and 1% Triton X-100 supplemented with protease inhibitors) and used as the ChIP input. Immunoprecipitation was done overnight at 4°C using GFP-Trap beads to capture GFP–FAN1 forms or an affinity-purified FAN1 sheep polyclonal antibody and protein G magnetic beads (20 μl of either bead per reaction). The isolated ChIP fractions were washed twice in Wash Buffer 1 (20 mm Tris–Cl, pH 8.1, 50 mm NaCl, 2 mm EDTA, 1% Triton X-100, 0.1% SDS), once in Wash Buffer 2 (10 mm Tris–Cl, pH 8.1, 150 mm NaCl, 1 mm EDTA, 1% NP40 and 1% Na deoxycholate) and once in Tris–EDTA. Bound material was eluted at 65°C for 30 min in 1% SDS and sodium 0.1 m NaHCO_3_. Cross links were reversed in ChIP and input fractions by adding NaCl to 250 mm and proteinase K (PK) (2 μl of 0.5 mg/ml PK per reaction) and heating at 65°C for at least 4 h. DNA was purified using columns and subjected to PCR using the following primers (genomic positions are given in brackets): HTT con 1 forward TTTGCCCAGGGAATCTTTGC, reverse TTGCAAGCGGAGAGAGAAGA (25886-26037); HTT con 2 forward TGCCTTTCGAAGTTGATGCA, reverse TGCCACCACGAATTTCACAA (137990-138191); HTT CAG 1 forward GAGTCCCTCAAGTCCTTCCAGCA, reverse CTGAGGAAGCTGAGGAGGC (179-309); HTT CAG 2 forward GAGTCCCTCAAGTCCTTCCAGCA, reverse GCCCAAACTCACGGTCGGT (179-426); TBP control forward AAGAGTGTGCTGGAGATGCT, reverse ATGCCCTTCCTTGCCTTTTG (2605-2831); TBP CAG forward CAGCCAGCCTAACCTGTTTT, reverse CTGCCTTTGTTGCTCTTCCA (7421-7577); ATXN3 control forward TATCCGTCTTGCAAGGTGGT, reverse CCCTGAATTGACGGCAGATG (7359-7534); ATXN3 CAG forward TTCAGACAGCAGCAAAAGCA, reverse AAAGTGTGAAGGTAGCGAACAT (35564-35732); DMPK control forward TGGGCCCAAAGACTCCTAAG, reverse TCTGAAGTCCTGTGGCTCTG (12722-12893); and FXN control forward AAGCGTGCATTTTGGATTCAA, reverse TTTTCAATTCCCTCACTGTCCTT (24300-24488). Quantification was done using Sybr Green reagents and a QuantStudio real-time PCR machine and software. MSN gene expression was assayed using Taqman Fast Advanced Master Mix and probes (ThermoFisher, Scientific, UK). Data were analyzed using the 2–∆∆Ct method, with ACTB, ATP5B, EIF4A2 and SDHA as housekeeping genes and normalized to the control 109Q iPSC line.

### Western blot and immunoprecipitation

Cell extracts were prepared for SDS-polyacrylamide gel electrophoresis (PAGE) as described previously (44). The antibodies used were a FAN1 sheep polyclonal antibody generated for this study; MSH3 or MLH1 monoclonal antibodies (BD Biosciences, UK); HTT exon 1-specific monoclonal antibodies 4C9 (Millipore, Merck-Millipore, UK) and MSH2 (Cell Signaling Technology, Danvers, MA, USA); and PMS2, GAPDH and GFP rabbit polyclonal antibodies (Santa Cruz Biotechnology, Dallas, TX, USA). Immunoblots were quantified with the Odyssey CLx Imaging System, (Lincoln, NE, USA) using Glyceraldehyde 3-phosphate dehydrogenase (GAPDH) and β-actin as loading controls. For Immunoprecipitation (IP) analysis, washed cells were resuspended in IP buffer (20 mm Tris, pH 7.4, 150 mm NACl, 1 mm EDTA, 1% Triton X-100 supplemented with Benzonase 2 U/ml and protease inhibitors) and incubated on ice for 20 min. The cell extracts were centrifuged at 10 000 *g* for 2 min and the supernatant fraction was used as input. GFP-Trap beads or the FAN1 goat polyclonal and MSH3 or MSH2 monoclonal antibodies and protein G magnetic beads were used to capture protein complexes. Beads were washed 3 times in IP buffer and eluted by heating in SDS sample buffer.

### Statistical analysis

Curve estimation was performed in SPSS (IBM) by producing regression statistics for 11 different models. Variance from the model and significance was measured by analysis of variance. Expansion rate was calculated from the slope of linear regression model fitted to change in modal CAG repeat length and was expressed as number of days per change in modal CAG length (±95% confidence interval). Expansion rates of treated or empty vector cells were compared with control cells by analysis of variance between the slopes of the linear regression lines in SPSS (IBM).

### Testing for differences in rate of CAG expansion between treatments

The rate of CAG expansion was modeled as a linear regression of CAG expansion on the number of days since the start of treatment. Differences in rate of CAG expansion between treatments were modeled by the inclusion of a days*treatment interaction term in the regression, and the significance of this term determines the significance of CAG expansion rate differences between treatments. Correlations between multiple CAG expansion measurements from the same replicate cell line were modeled by including cell line-specific random effects on CAG expansion rate in the analysis. Models were fitted using the lmer function in R.

Initially, global differences in CAG expansion rate were tested between all treatments simultaneously. If this test was significant, post-hoc tests were performed to characterize the differences.

## Supplementary Material

Supplementary Data
