# Supplementary material for: FAN1 modifies Huntington’s disease progression by stabilizing the expanded HTT CAG repeat
Source: Hum Mol Genet. 2018 Oct 24;28(4):650–61. doi: 10.1093/hmg/ddy375 (PMC6360275; doi:10.1093/hmg/ddy375)
Supplement: Supplementary Data [file ddy375_supp.zip › Goold et al 2018_Supplementary Material.pdf]

Supplementary Material

Figure S1. Characterisation of FAN1 variant activity

(A) Immunoblot showing FAN1 levels in U20S cells reconstituted with WT or p.R507H variant GFP-FAN1. Parental line expressing endogenous $FAN1^{+/+}$ and the $FAN1^{-/-}$ line are shown for comparison. Note the slowed mobility of the GFP-FAN1 forms. **(B)** GFP immunofluorescence shows nuclear expression of the GFP-FAN1 forms. WT and p.R507H variant FAN1 form DNA repair foci following MMC treatment (100ng/ml for 2h). **(C)** Extracts from U20S cells expressing GFP-FAN1 WT, p.R507H variant or $FAN1^{-/-}$ cells were prepared and subjected to IP with GFP-Trap beads. Input (In) and immunoprecipitate fractions (IP) were separated by SDS PAGE and Western blots were probed with the indicated antibodies. FAN1 IP fractions from cells expressing GFP-FAN1 WT and p.R507H contained readily detectable MLH1, PMS2 and FANCD2. These proteins were not detected in control IP fractions from $FAN1^{-/-}$ cells, indicating the specificity of the IP procedure. **(D)** U20S cell lines were treated with MMC at various concentrations for 16 h. The cells were washed into fresh media and viability was assayed after 10 days in culture. $FAN1^{-/-}$ cells are vulnerable to MMC toxicity. Endogenous $FAN1^{+/+}$, GFP-WT and GFP-p.R507H variant FAN1 infer protection. (n = 3, ± SEM). **(E)** WT and p.R507H variant FAN1 clear H2AX foci following Cisplatin treatment (1 μg/ml, for 2 h). **(F)** Immunoblot showing FAN1 levels in HD-LB cells line expressing WT FAN1 or p.R507H variant FAN1 (heterozygous). GAPDH was used as a loading control. $FAN1^{-/-}$ run alongside the LB extracts demonstrate the specificity of the FAN1 antibody. **(G)** WT and p.R507H LB cell lines were treated with MMC at various concentrations for 16 h. The cells were washed into fresh media and viability was assayed after 10 days in culture(n = 3, ± SEM). **(H)** Immunoblot showing IP fractions from extracts prepared from $FAN1^{WT}$ LB cells. Input (In - 5%), immunoprecipitate (IP) and the unbound fraction (IPs) are shown

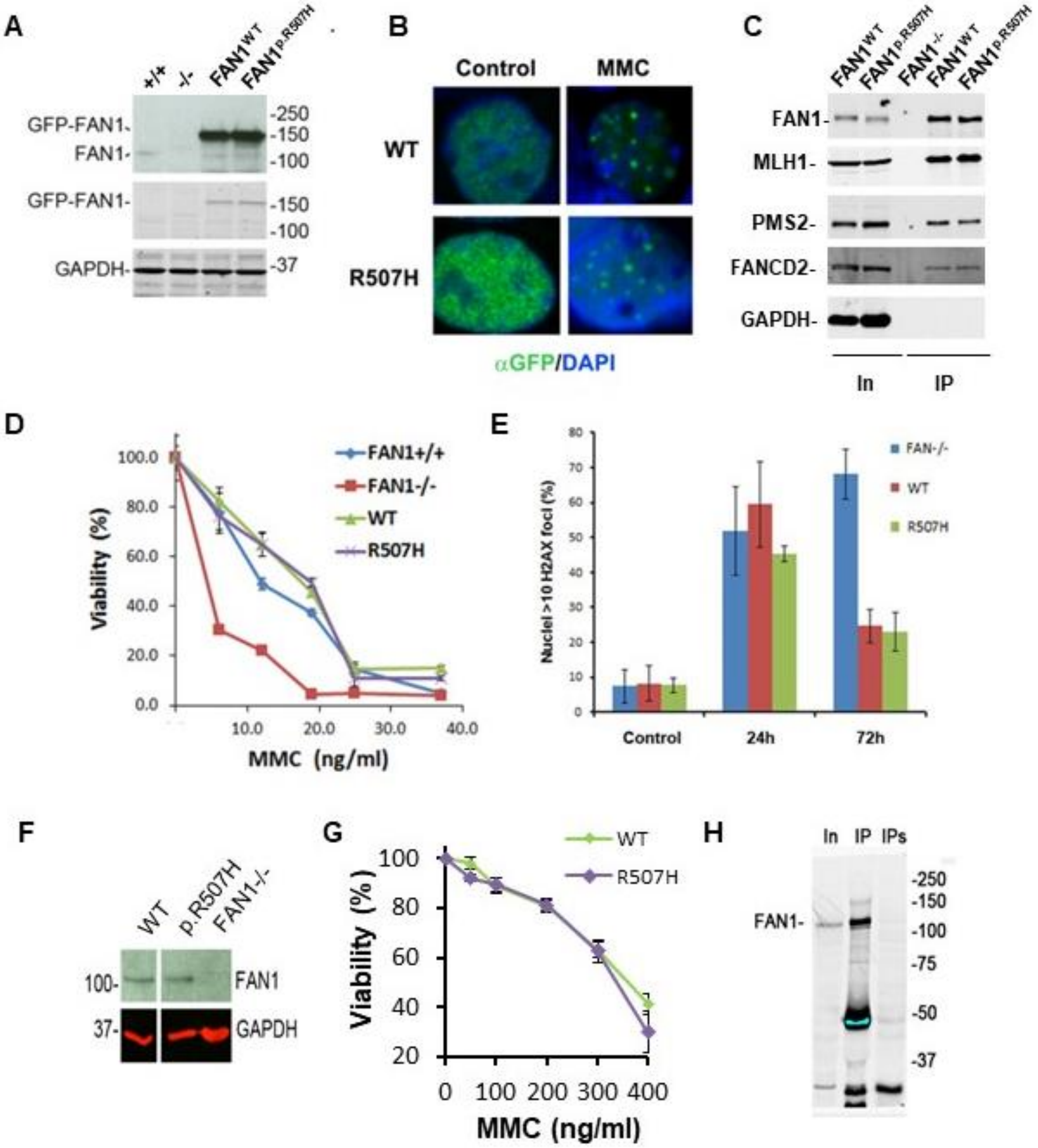
A
+/+
-/-
FAN1WT
FAN1p.R507H
GFP-FAN1-
FAN1-
-250
-150
-100
GFP-FAN1-
-150
-100
GAPDH-
-37
B
Control
MMC
WT
R507H
αGFP/DAPI
C
FAN1WT
FAN1p.R507H
FAN1-/-
FAN1WT
FAN1p.R507H
FAN1-
MLH1-
PMS2-
FANCD2-
GAPDH-
In
IP
D
FAN1+/+
FAN1-/-
WT
R507H
Viability (%)
MMC (ng/ml)
E
FAN-/-
WT
R507H
Nuclei >10 H2AX foci (%)
Control
24h
72h
F
WT
p.R507H
FAN1-/-
100-
FAN1
37-
GAPDH
G
WT
R507H
Viability (%)
MMC (ng/ml)
H
In
IP
IPs
FAN1-
-250
-150
-100
-75
-50
-37

Figure S2. Characterisation of the CAG repeat stability in 73 and 109 CAG iPSC lines

(A) An iPSC line with 73 CAG repeats was cultured as mitotic cells for 80 days or as differentiated medium spiny neurons (MSN) for 193 days (differentiation was initiated at day 0, MSNs form a large proportion of the cells by day 35). Over time in culture there was no expansion of the CAG repeat in either as iPSCs or MSNs (p = 0.61 as iPSC and p = 0.66 as MSN, slope non-zero).**(B)** Long term culture reveals 109 CAG iPSC repeat expansion fits to an exponential model ($p = 7.65x10^{-26}$, $y = 121.1e^{0.0005x}$, $R^2 = 0.9887$).

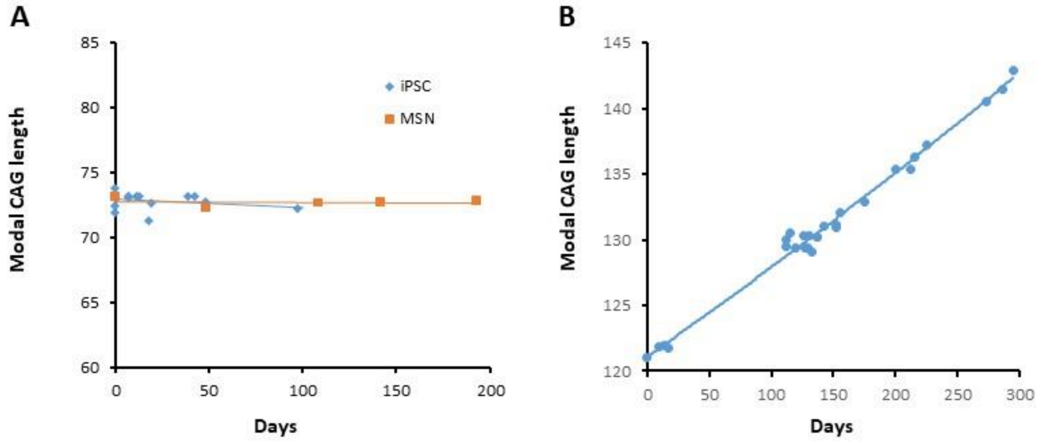

Figure S3. Expression of MSN specific striatal genes following differentiation of 109 CAG iPSCs

Expression of a panel of MSN specific striatal genes, is significantly increased in differentiated MSNs relative to iPSC. Fold change is shown relative to control 109Q iPSC cells, analyses performed in triplicate, error bars represent standard error of the mean.

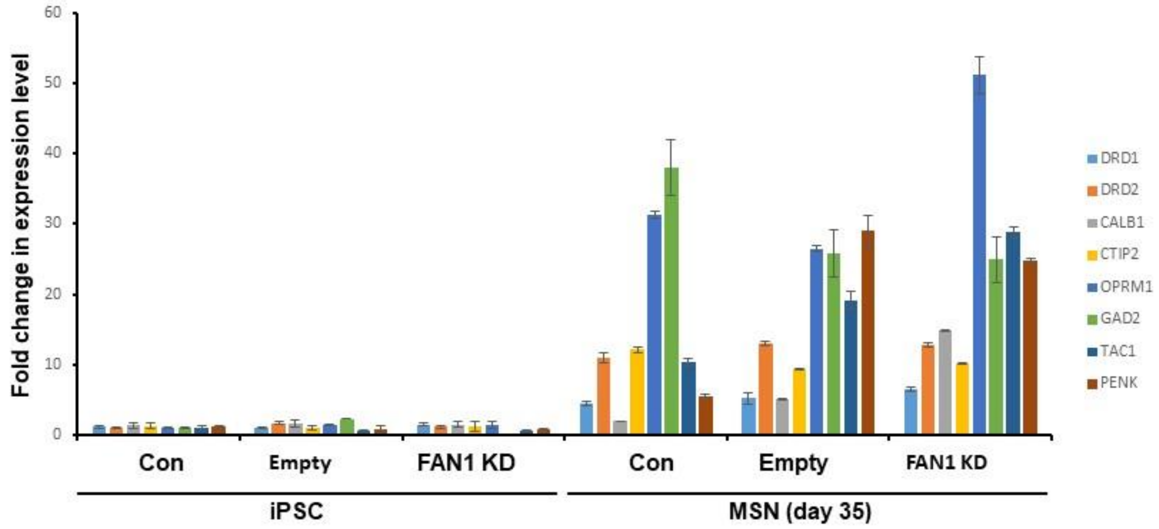

Figure S4. ChIP analysis suggests FAN1 WT and p.R507H variant interacts with the expanded CAG repeat region of *HTT*

(A) Extracts from U20S cells expressing GFP-FAN1 WT or p.R507H and transiently transfected with *HTT* exon 1 (118 CAG) were prepared for ChIP analysis. GFP-Trap magnetic beads were used to isolate FAN1 and associated DNA. $FAN1^{-/-}$ cells or untransfected U20S cells expressing GFP-FAN1 WT were used as a control. Primers spanning the CAG repeat of the *HTT* gene were used to amplify isolated DNA. PCR products were visualised on an agarose gel. CAG primers amplify DNA from the input fractions derived from the cells transfected with *HTT* exon 1 (118 CAG) (5 % input was used as a PCR substrate). ChIP fractions derived from cells expressing FAN1 (WT or p.R507H variants) were amplified by primers spanning the CAG repeat region. Note, ChIP fractions from $FAN1^{-/-}$ cells or untransfected cells did not contain detectable amounts of DNA showing the specificity of the ChIP procedure. Note the PCR conditions used to amplify DNA from the introduced exon 1 construct, present in high copy numbers, did not detect endogenous *HTT* gene. (**B**-**C)** Genomic DNA and protein lysates were prepared from U20S *HTT* exon1 (70 CAG), *HTT*.exon1 (97 CAG) and *HTT* exon1 (118 CAG) FAN1 knockout (-/-) cells **(B)** PCR across the CAG repeat showed incorporation of the expanded *HTT* exon 1 transgenes and the endogenous gene (PCR products between 400-600 bp) and the endogenous gene (20 CAG repeats, a PCR product ~200 bp in all samples). **(C)** Western blot probed with 4C9 and anti-GFP antibodies show the inserted *HTT* exon 1 transgenes are transcriptionally active **(D)** Extracts from HD 109 CAG iPSC cells homozygous for $FAN1^{WT}$ were prepared for ChIP analysis and a FAN1 antibody was used to isolate FAN1 and associated DNA. Mock pull downs with no antibody were used as controls. Primers spanning the CAG repeat of the *HTT* gene or control primers recognising DNA downstream of the repeat (HHT 2) were used to amplify isolated DNA. PCR products were visualised on an agarose gel. Input (5%) and ChIP fractions are shown. The two bands seen in the input and ChIP fractions are amplified DNA from normal and mutant expanded *HTT* alleles. No antibody controls contained little DNA showing the specificity of the ChIP procedure. **(E)** ChIP fractions shown in D were amplified with primer pairs recognizing various regions of *HTT* (CAG repeat and non CAG regions – HTT 1 and 2). No significant differences in the levels of the amplified DNA from different HTT regions were detected.

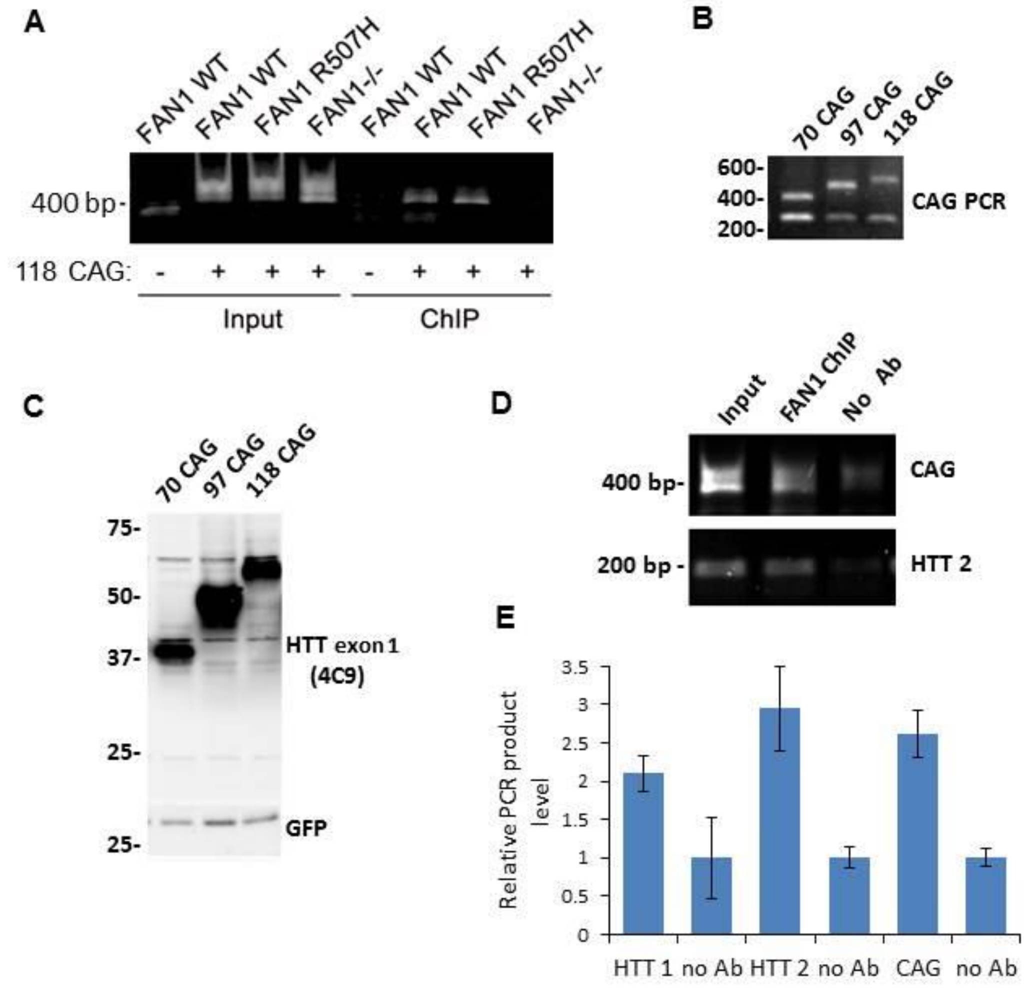
A
FAN1 WT
FAN1 WT
FAN1 R507H
FAN1-/-
FAN1 WT
FAN1 WT
FAN1 R507H
FAN1-/-
400 bp
118 CAG: - + + + - + + +
Input
ChIP
B
70 CAG
97 CAG
118 CAG
600-
400-
200-
CAG PCR
C
70 CAG
97 CAG
118 CAG
75-
50-
37-
25-
25-
HTT exon 1
(4C9)
GFP
D
Input
FAN1 ChIP
No Ab
400 bp-
200 bp -
CAG
HTT 2
E
Relative PCR product level
3.5
3
2.5
2
1.5
1
0.5
0
HTT 1
no Ab
HTT 2
no Ab
CAG
no Ab
